# Marine Group II Archaea, potentially important players in the global ocean carbon cycle

**DOI:** 10.3389/fmicb.2015.01108

**Published:** 2015-10-13

**Authors:** Chuanlun L. Zhang, Wei Xie, Ana-Belen Martin-Cuadrado, Francisco Rodriguez-Valera

**Affiliations:** ^1^State Key Laboratory of Marine Geology, Tongji UniversityShanghai, China; ^2^Department of Producción Vegetaly Microbiología, Universidad Miguel HernándezAlicante, Spain

**Keywords:** archaea, Marine Group II, heterotrophic metabolism, carbon cycle

## Abstract

Marine Group (MG) I (currently known as *Thaumarchaeota*) and MG II *Archaea* were first reported over two decades ago. While significant progress has been made on MG I microbiology and ecology, the progress on MG II has been noticeably slower. The common understanding is that while MG I mainly function as chemolithoautotrophs and occur predominantly in the deep ocean, MG II reside mostly in the photic zone and live heterotrophically. Studies to date have shown that MG II are abundant in the marine aquatic environment and display great seasonal and spatial variation and phylogenetic diversity. They also show unique patterns of organic carbon degradation and their energy requirements may be augmented by light in the photic zone. However, no pure culture of MG II has been obtained and thus their precise ecological role remains elusive.

## Introduction

In 1992, DeLong and Fuhrman et al. reported the occurrence of archaea in the cold ocean. Two phylogenetic groups were described based on 16S rRNA sequences: Marine Group I (MG I) within the *Crenarchaeota* and Marine Group II (MG II) within the *Euryarchaeota*. Before this discovery, archaea were considered to be obligate extremophiles. Subsequent cultivation-independent microbial surveys revealed them to be abundant and widespread in relatively “common” environments like soil or the most extensive habitat on Earth, the oligotrophic ocean.

Over the past 22 years, tremendous progress has been made on characterizing MG I, which have been reclassified as the archaeal phylum *Thaumarchaeota* (Brochier-Armanet et al., 2008). However, our understanding of MG II remains fragmented and significantly less than MG I. In contrast to MG I, no pure cultures or even enrichments are available for MG II. Breakthroughs have been made, however, in understanding the potential physiology and biochemistry of MG II by metagenomic approaches (Iverson et al., 2012; Deschamps et al., 2014; Martin-Cuadrado et al., 2015; Orsi et al., 2015). This mini-review summarizes our current understanding of this group and provides an outlook for future research on MG II.

## Abundance and distribution of MG II and other *Euryarchaeota* in temperate latitudes

Early observations on the distribution of MG II were based on the relative abundance of clones in clone libraries generated from 16S rRNA genes amplified using PCR. In general, MG II contributed more to microbial assemblages from surface than deeper waters (Massana et al., 1997) while MG I were found to have more clones in deeper samples, which led to the conclusion that MG II dominate the photic zone and MG I the meso- and bathypelagic waters (Massana et al., 1997, 2000).

The first reliable quantitative analysis of their distribution was provided by fluorescence *in situ* hybridization (FISH), using either oligo-probes (oligoFISH) or poly-probes (polyFISH). The former tended to give higher estimates of the abundance of all archaea than the sum of MG I and MG II determined by polyFISH (Pernthaler et al., 2002) (Table 1). MG II were estimated to represent around 15% of total archaeal cells in the Atlantic Ocean, with little variation with depth (Teira et al., 2004). In contrast, a time series assessment of planktonic archaea in the Santa Barbara Channel revealed “intermittent” blooms of MG II coinciding with decreases in chlorophyll *a* (Murray et al., 1999). Another seasonal study of surface water at the German Bight in the North Sea showed a spring bloom of MG II, when they could be >30% of total cell counts and >90% of all archaeal cells (Pernthaler et al., 2002). Catalyzed reporter deposition-FISH (CARD-FISH) using specific oligonucleotide probes improved the quality of FISH quantification (Teira et al., 2004; Herndl et al., 2005) (Table 1). For example, Herndl et al. (2005) reported that the CARD-FISH method yielded twice as many euryarchaeotal cells than the oligoFISH method.

**Table 1:** **Abundance and distribution of Marine Group II in temperate latitudes and polar oceans**.

**Region**	Water depth (m)^*^	**Biotope**	**Type of euryarchaeota**	**Abundance (FISH or qPCR)**	**Note**	**References**
**TEMPERATE LATITUDES**						
Santa Barbara Channel	<100	Coastal	MG II	ND^§^	First description of MG II as mesophic, aerobic members of archaea	DeLong, 1992
Santa Barbara Channel	533	Coastal	MG II	10^4^ − 10^5^ cells/ml (OligoFISH)	MG II dominate over MG I in surface water	Massana et al., 1997
Northeast Pacific	500–3000	Open ocean	MG II, MG III	ND		Fuhrman and Davis, 1997
Subtropic Atlantic	1000	Open ocean	MG II	ND		Fuhrman and Davis, 1997
Monterey Bay, CA	450–4424	Offshore	MG II	10^4^ − 10^5^ cells/ml (PoliFISH)	MG II dominate over MG I in surface water	DeLong et al., 1999
Santa Barbara Channel	300	Coastal	MG II	ND	“Intermittent” blooms of MG II in surface water (<20 m)	Murray et al., 1999
North Atlantic Ocean	2850	Open ocean	MG II	ND	MG II dominate over MG I in surface water	Massana et al., 2000
Cantabrian Sea (Atlantic Ocean)	132	Slope	MG II	ND		Massana et al., 2000
Alboran Sea (Mediterranean Sea)	941	Open ocean	MG II, MG III	ND	MG II dominate over MG I in surface water	Massana et al., 2000
Santa Barbara Channel	522	Coastal	MG II	ND	MG II dominate over MG I in surface water	Massana et al., 2000
North Pacific subtropical gyre	4750	Open ocean	Total Euryarchaeota	<10% of total DAPI counts ~ 10^3^ − 10^4^ cells/ml	First seasonal quantification of archaea in the open ocean	Karner et al., 2001
Coastal North Sea (German Bay)	1	Coastal	Total Euryarchaeota	Euryarchaeota can be >30% of total picoplankton	PolyFISH is better than OligoFISH; observed spring bloom of MGII	Pernthaler et al., 2002
Monterey Bay, CA	1097	Coastal	Total Euryarchaeota	Up to 12% of DAPI counts in summer and <1% in winter		Pernthaler et al., 2002
North Atlantic Ocean	4000	Open ocean	Total Euryarchaeota	14–30% of DAPI counts		Teira et al., 2004
Atlantic Ocean	88–3869	Open ocean	MG II	10^3^ − 10^4^ cells/ml	Improved quatification using CARD-FISH	Herndl et al., 2005
North Atlantic Ocean	100–4000	Open ocean	MG II	16–18% of DAPI counts	Improved quatification using CARD-FISH	Teira et al., 2006
Southern North Sea	<50	Coastal	MG II	2 × 10^4^ cells/ml	Spring and summer blooms	Herfort et al., 2007
Gulf of Mexico	>800	Open ocean	MG II	ND	MG II dominate the surface-mid-depth waters	Liu et al., 2009
South China Sea	2418–3839	Open ocean	MG II	2–16% of total DAPI counts	MG II dominate the surface water	Zhang et al., 2009
Mediterranean Sea	20	Coastal	MG IIa, MG IIb, MG III	Relative sequence abundance	First observation of seasonal differentiation within MG II: MG IIa (winter), MG IIb (summer)	Galand et al., 2010
Coastal northwest Mediterranean Sea	3	Coastal	MG IIa, MG IIb	Relative sequence abundance	Same conclusion as above	Hugoni et al., 2013
Pacific Ocean	>500	Open ocean	MG II	Up to 4 × 10^5^ cells/l^∫^	MG II outnumber MG I throughout the water column in large particle (3- to 57 - μm) fractions	Lincoln et al., 2014a
**POLAR OCEANS**						
Coastal Antarctica	0	Coastal	Total Euryarchaeota	>60% of total archaea	Estimated based on the difference between total archaea and MG I	DeLong et al., 1994
Coastal Antarctica	0	Coastal	MG II	ND		DeLong et al., 1998
Coastal Antarctica	<50	Coastal	MG II	10^4^ cells/ml	MGII is minor fraction of total archaeal plankton (3–40 m) (under sea ice)	Murray et al., 1998
Antarctica Peninsula	500–1000	Strait	MG II	ND		Massana et al., 1998
Antarctic polar front	3000	Strait	MG II, MG III, MG IV	Exclusively euryarchaeotal species		López-Garcìa et al., 2001a
Antarctic polar front	3000	Strait	MG II, MG III, MG IV	ND		López-Garcìa et al., 2001b
West of Antarctic Peninsula	200–500	Shelf	MG II	10^4^ cells/ml		Church et al., 2003
Antarctic polar front	500	Strait	MG II	ND		Moreira et al., 2004
Canadian Archipelago	7–600	Archipelago	Total archaea	ND	Archaea correlate significantly with particle concentrations	Wells and Deming, 2003
Arctic Ocean/Antarctic	55–235	Nearshore to offshore	MG II, MG III, MG IV	Equal abundance in MGI and MGII	No seasonal variation, but variation occurs with depth	Bano et al., 2004
Beaufort Shelf/Franklin Bay	0–150	Coastal	MG II	ND		Wells et al., 2006
Beaufort Shelf	5–33	Coastal	MG II	ND		Galand et al., 2006
Western Arctic Ocean	0 ≥ 500	Shelf to basin	MG II	<10% of total DAPI counts		Kirchman et al., 2007
Western Arctic Ocean	3–100	Coastal	MG II	<5% of total DAPI counts		Alonso-Sáez et al., 2008
Beaufort Shelf	5–260	Nearshore to offshore	MG II	ND		Galand et al., 2009a
North Water (Arctic)	62–180	Nearshore to offshore	MG II	10^4^–10^5^ copies/mL	MG IIa dominates	Galand et al., 2009b
Arctic Ocean	50–3820	Offshore	MG II, MG III, MG IV	Dominance of MGIII in deep water		Galand et al., 2008

* *Bottom water depth or max sampling depth*.

§ *Not determined using either FISH or qPCR*.

∫ *Inferred from difference between MGI abundance based on qPCR and archaeal community composition in metagenomic datasets*.

Quantitative PCR (qPCR) was used to estimate the relative abundance of different archaea in surface waters of Blanes Bay in the NW Mediterranean Sea (Galand et al., 2010). This was the first multiple year study showing seasonal variation within MG II, which was divided into two distinct lineages, II.a and II.b, with MG II.b being more abundant during winter mixing when nutrients are more abundant and MG II.a being predominant in summer when nutrients become depleted (Galand et al., 2010) (Table 1). This study led to a better understanding of the distinct ecological roles of MG II in this region and revealed seasonal variation in activity levels or growth rates that are possibly driven by different metabolism and life strategies (Hugoni et al., 2013).

## Abundance and distribution of MG II and other *Euryarchaeota* in polar oceans

Early studies of planktonic archaea in polar oceans were carried out in surface water near Arthur Harbor on the Antarctic Peninsula. These studies revealed that planktonic archaea could count for up to 34% of the prokaryotic cell abundance (DeLong et al., 1994) (Table 1). Later studies (Murray et al., 1998; DeLong et al., 1999) in the same region reported the predominance of MG I over MG II in winter surface water. Sampling in a more dynamic aquatic system in the center of the Gerlache Strait on the Antarctic Peninsula revealed varying contribution of MG II to total planktonic archaea at different depths, with a larger fraction of MG II contributing to the archaeal assemblage at the surface than at depth (Massana et al., 1998).

A comprehensive polyFISH approach was applied to examine the waters off the Antarctic Peninsula for vertical and temporal changes in archaeal cell abundance (Church et al., 2003). The results showed that MG II was low in abundance (<10% of total picoplankton) throughout the water column and did not differ significantly between summer and winter (Church et al., 2003). Based on these results and those of others (DeLong et al., 1998; Massana et al., 1998, 2000; Murray et al., 1999), Church et al. (2003) concluded that MG II were not numerically abundant in the plankton communities of the Southern Ocean. The occurrence of marine MG III and MG IV, two new lineages of *Euryarchaeota* that are closely related to MG II, has also been observed in deep waters of the Antarctic Polar Front (López-Garcìa et al., 2001a,b).

Bano et al. (2004) were the first to report on the occurrence of MG II, MG III, and MG IV in the Arctic Ocean (Table 1). They also made a comparison of archaeal distribution between Arctic and Antarctic waters and observed that some *euryarchaeotal* ribotypes were unique to each system. Galand et al. (2006) further observed that MG II were actually the most common archaeal group in 16S rRNA gene clone libraries constructed from the coastal Beaufort Sea. They speculated that the greater abundance of MG II in the Arctic Ocean may be related to the higher availability of labile organic matter from land surrounding the Arctic, as demonstrated by the significant impact of terrestrially derived particles on coastal archaea in the Mackenzie River-influenced Beaufort Sea (Galand et al., 2006, 2008; Wells et al., 2006). *Euryarchaeotal* abundance was very low throughout the year in surface waters of the Western Arctic (Kirchman et al., 2007; Alonso-Sáez et al., 2008) (Table 1), which lacked direct river inputs (Galand et al., 2008).

## Diversity and phylogeny of MG II and other *Euryarchaeota*

Early studies using 16S rRNA gene phylogenies showed limited diversity of the MG II group and placed it in close association with *Thermoplasma* or methanogens (DeLong, 1992; Fuhrman et al., 1992; Massana et al., 1997, 2000). Nevertheless, two major clusters within MG II (which became the MG II.a- and MG II.b lineages later on) and a third branch (MG III) within the *Euryarchaeota* were also identified in these studies (Fuhrman et al., 1992; Massana et al., 2000).

Our knowledge of MG II diversity was enhanced by the application of high throughput sequencing and metagenomic analysis to studies of bacterial community composition, which revealed expansive diversity of MG II in coastal or estuarine waters. For example, Bano et al. (2004) observed that certain MG II sequences were predominately found in the surface water, the mixed-layer, or the halocline in the Arctic Ocean. Similarly, Galand et al. (2009a) observed a higher diversity of MG II in Arctic surface water in comparison to MG III that were more diverse in deeper Arctic water. Liu et al. (2009) observed that MG II had the greatest diversity in surface water, which decreased with depth in the Gulf of Mexico. Lincoln et al. (2014a) reported that MG II contributed a much higher proportion to total archaeal operational taxonomic units (OTUs) at shallower depths in the Pacific Ocean; however, different individual OTUs dominated the MG II population at different depths. The predominance of MG II in surface water and MG III in deep water was also observed in the South China Sea (Tseng et al., 2015). Overall, MG I share >94% 16S rRNA gene sequence similarity, whereas MG II only share 85% (Massana et al., 2000; Bano et al., 2004; Herfort et al., 2007), hence, MG II appear to be more phylogenetically diverse than MG I.

## Genomics and metagenomics of MG II and their predicted physiology

The distributions and abundances of MG II ribotypes (as well as MG III or MG IV) indicate that planktonic *Euryarchaeota* occupy diverse ecological niches (Murray et al., 1999; Hugoni et al., 2013; Lincoln et al., 2014a). However, little is known about the underlying mechanisms of niche partitioning among these organisms. The differences in distributions of MG II.a and MG II.b during different seasons observed in the Mediterranean Sea were attributed to differential sensitivity to temperature or nutrient and oxygen availability (Hugoni et al., 2013). Overall, our understanding of the ecological and biogeochemical functions of MG II is very fragmentary and incomplete, largely due to the lack of pure cultures and whole genomes that would allow us to better study the physiology and biochemistry of these organisms. However, information from metagenomics and reconstructed or partially assembled genomes is providing insights about these microbes.

Light-harvesting capability of MG II was first deduced from genomic fragments containing proteorhodopsins (Frigaard et al., 2006). So far only MG II from the photic zone are found to contain the proteorhodopsin gene, which occurs in about 10% of the euryarchaeal population (Frigaard et al., 2006). It has been suggested that proteorhodopsins could support a photoheterotrophic lifestyle by generating a light-driven chemiosmotic potential (Frigaard et al., 2006; Iverson et al., 2012). Summer peaks of abundance and activity of MG II.a in surface water of the Mediterranean Sea were attributed to the enhanced phototrophy in response to greater irradiance (Hugoni et al., 2013).

Despite the identification of proteorhodopsins in MG II genomes, Deschamps et al. (2014) did not find any genes encoding proteorhodopsin homologs in a MG II/III-*Euryarchaeota* dataset of nine metagenomes from deep-Mediterranean waters. Similarly, Baker et al. (2013) failed to detect any proteorhodopsin genes in the deep Guaymas metatranscriptome. These results support an earlier conclusion that deep-sea dwelling MG II *Euryarchaeota* are different from proteorhodopsin-containing MG II living in the ocean photic zone (Frigaard et al., 2006). Deschamps et al. (2014) also noted that the deep sea MG II *Euryarchaeota* have abundant genes targeting amino acid, carbohydrate and lipid transport and metabolism, which are typical of heterotrophic prokaryotes. Orsi et al. (2015) found that the rhodopsins of MG II in metagenomes from large cell size fractions (>0.8 μm) are phylogenetically distinct from rhodopsin genes found in metagenomes obtained with smaller size fractions, suggesting a possible difference in photoheterotrophy between the free-living and the particle-associated MG II groups.

Iverson et al. (2012) obtained the first nearly complete genome of a MG II, belonging to subgroup II.a, from metagenomic assemblies obtained from surface seawater in the Puget Sound. The genome suggested that MG II.a was a particle-associated microbe and the cells were predicted to be motile, photo-heterotrophic and capable of degrading polymers such as proteins and lipids. This validated the prediction of Béjà et al. (2000) that surface MG II were capable of proteolysis. The deep water genomic fragments (Moreira et al., 2004; Martin-Cuadrado et al., 2008; Deschamps et al., 2014) do not seem to have this metabolic feature. More recently, Orsi et al. (2015) quantified the abundance and distribution of MG II 16S rRNA genes in size-fractionated seawater samples from the euphotic zone of the central California Current System and showed that MG II abundance was highest in the particulate fraction, indicating that some MG II euryarchaeotes were physically associated with particles. These authors also found that the genome content of particle-attached MG II suggested an increased capacity for surface adhesion, transcriptional regulation and catabolism of high molecular weight substrates.

Another genome of MG II was assembled from the Mediterranean deep chlorophyll maximum (DCM), which belonged to subgroup II.b (Martin-Cuadrado et al., 2015). The authors used FISH to detect these cells in DCM samples and proposed the Class *Thalassoarchaea* (archaea from the sea) to name members of the MG II.b. They also confirmed by recruitment of genomic fragments from the Mediterranean Sea and Puget Sound that *Thalassoarchaea* are inhabitants of the oligotrophic photic zone while MG II.a are adapted to more coastal or even estuarine (brackish) habitats, which indicate that subgroups of MG II may represent different ecotypes. Two recent metatranscriptomic studies (Baker et al., 2013; Ottesen et al., 2013) found that MG II transcripts were present at levels similar to numerically dominant groups like *Pelagibacter* or SAR86. Actually, metatranscriptomic data show in general much greater contribution of MG II than their expected numbers from metagenomics would predict, suggesting that MG II populations may be very dynamic and capable of responding rapidly to changing conditions.

**Figure 1:**
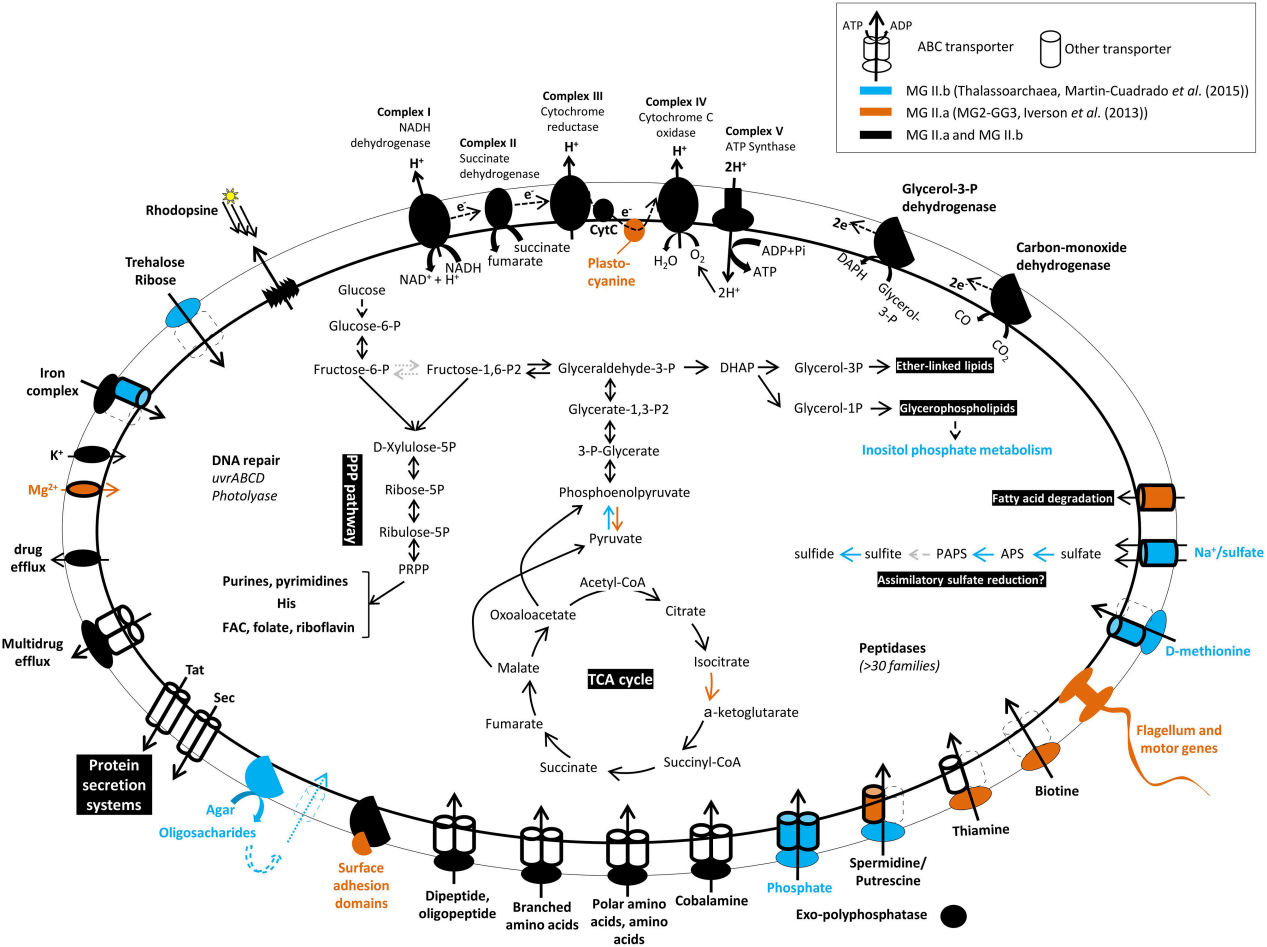
**Predicted metabolism of Marine Group II Archaea (groups II.a and II.b) based on genomes reconstructed from metagenomes available in the literature**.

A diagram summarizing the key metabolic functions of the MG II as derived from metagenomics is displayed in Figure 1. In general, MG II metabolic genes include those encoding functions associated with glycolysis, the tricarboxylic acid cycle, and phosphorylation complexes indicative of aerobic respiration. However, the identification of some of the genes of the assimilatory sulfate reduction pathway in the *Thalassoarchaea* genomic fragments (Martin-Cuadrado et al., 2008, 2015) suggests capacity for anaerobic respiration within low-oxygen microenvironments such as organic particles, which is supported by Orsi et al. (2015). In addition, a complete non-oxidative pentose phosphate pathway was identified in both available genomes of MG II, but some of the enzymes of the irreversible oxidative branch were not found in MG II.b, probably due to the incompleteness of this genome. Several transporters were identified, but the nature of the substrates could not be identified in most of the cases. An agarase-like gene is present in the MG II.b (Figure 1); however, it is unknown whether this group of archaea can use agar as carbon source. Transporters for branched-chain amino acids and di/oligopeptides were very abundant, supporting the model that protein degradation may be important to the metabolism of these microbes. Also, sequences encoding several drug-efflux pumps were abundant in both clades, which was suggested to indicate a defensive life style, typical of organisms exposed to natural toxin, i.e., from blooms of cyanobacteria producing marine biotoxins (Martin-Cuadrado et al., 2015).

Last but not least, the biogeochemical function of MG II is receiving increasing attention. The TEX_86_ paleo sea surface temperature proxy was originally based on the premise that archaeal lipids used in formulating the proxy are solely produced by *Thaumarchaeota* living in the surface ocean (Schouten et al., 2002). However, evidence increasingly demonstrates that temperature is not the only variable affecting the tetraether lipid distribution in the ocean (see reviews by Pearson and Ingalls, 2013; Schouten et al., 2013). In particular, the biological source of tetraether lipids in the ocean requires further consideration. Recently, Lincoln et al. (2014a) and Wang et al. (2015) provide evidence supporting an earlier speculation (Turich et al., 2007) that MG II may be significant contributors to tetraether lipids in coastal and open oceans. This is, however, still a highly debated topic (Lincoln et al., 2014b; Schouten et al., 2014) and a critical step and potentially growing area of research is verifying tetraether production by MG II under controlled laboratory conditions, using either enrichment or, hopefully, pure cultures.

## Summary and future research directions in marine archaea

Archaea are now recognized as equally important as bacteria in the global ocean carbon cycle. Advances in MG I (*Thaumarchaeota*) research over the past two decades have been tremendous, particularly in our understanding of the ecological and biogeochemical functions of these organisms. In comparison, our understanding of MG II, despite their widespread occurrence in the ocean, is still rudimentary. Recently, the concept of the microbial carbon pump (Jiao et al., 2010) further highlighted the importance of microorganisms (including both archaea and bacteria) in long term storage of dissolved organic carbon, which is the largest pool of organic carbon in the ocean. While a number of issues hinder advancing our understanding of the microbial processes mediating ocean biogeochemistry, the lack of information on archaea is particularly severe (Kujawinski, 2011). Archaea have several unique features that distinguish them from bacteria, including membrane lipids that are more resistant to degradation than bacterial lipids and capability in surviving harsh environments in which carbon metabolism by other organisms may be inhibited. Future research on marine archaea, including both MG I and MG II, may focus on the following topics in order of importance and ease of accomplishment for the fields of microbial ecology and biogeochemistry:

1. Increasing genomic coverage of MG II lineages using metagenomics and single-cell genomics.
2. Enriching and isolating MG II followed by physiological and biochemical studies.
3. Validating the lipid composition of MG II and re-evaluating the sources of GDGTs in the open ocean.
4. Distinguishing relative contributions of archaea and bacteria in the production and transformation of recalcitrant dissolved organic carbon in the ocean.
5. Elucidating the evolutionary and/or horizontal gene transfer pathways of MG II in contrast to or in concert with MG I.

### Conflict of interest statement

The authors declare that the research was conducted in the absence of any commercial or financial relationships that could be construed as a potential conflict of interest.
